# Chromosome abnormalities in Indonesian patients with short stature

**DOI:** 10.1186/1755-8166-5-35

**Published:** 2012-08-06

**Authors:** Chrysantine Paramayuda, Hannie Kartapradja, Debby D Ambarwati, Helena W Anggaratri, Lita P Suciati, Nanis S Marzuki, Alida Harahap

**Affiliations:** 1 Eijkman Institute for Molecular Biology, Jakarta, Indonesia

**Keywords:** Short stature, Chromosome abnormalities, Low-level mosaicism, FISH

## Abstract

**Background:**

Short stature is associated with several disorders including wide variations of chromosomal disorders and single gene disorders. The objective of this report is to present the cytogenetic findings in Indonesian patients with short stature.

**Methods:**

G-banding and interphase/metaphase FISH were performed on short stature patients with and without other clinical features who were referred by clinicians all over Indonesia to our laboratory during the year 2003–2009.

**Results:**

The results of chromosomal analysis of ninety seven patients (mean age: 10.7 years old) were collected. The group of patients with other clinical features showed sex chromosome abnormalities in 45% (18/40) and autosomal abnormalities in 10% (4/40), whereas those with short stature only, 42.1% (24/57) had sex chromosome abnormalities and 1.75% (1/57) had autosomal abnormalities. The autosomal chromosomal abnormalities involved mostly subtelomeric regions. Results discrepancies between karyotype and FISH were found in 10 patients, including detection of low-level monosomy X mosaicism in 6 patients with normal karyotype, and detection of mosaic aneuploidy chromosome 18 in 1 patient with 45,XX,rob(13;14)(q10;q10).

Statistical analysis showed no significant association between the groups and the type of chromosomal abnormalities.

**Conclusion:**

Chromosome abnormalities account for about 50% of the short stature patients. Wide variations of both sex and autosomal chromosomes abnormalities were detected in the study. Since three out of five patients had autosomal structural abnormalities involving the subtelomeric regions, thus in the future, subtelomeric FISH or even a more sensitive method such as genomic/SNP microarray is needed to confirm deletions of subtelomeric regions of chromosome 9, 11 and 18. Low-level mosaicism in normal karyotype patients indicates interphase FISH need to be routinely carried out in short stature patients as an adjunct to karyotyping.

## Introduction

Short stature is defined as a standing height below the 3^rd^ percentile for sex according to standard growth chart [1,2] and is believed to have a strong genetic component. There are various genetic causes of short stature, which include chromosomal defects. It has been known that short stature and poor growth is associated with Turner syndrome, Down syndrome and other syndromes [2,3]. Therefore, chromosome and molecular analysis are frequently required in short stature cases.

Haploinsufficiency of SHOX gene, which located at short arm of X chromosome, is reported to be responsible for height deficit in Turner syndrome patients and other short stature patients, including isolated short stature without dysmorphic features, Leri-Weill syndrome, or Langer syndrome [4]. Furthermore, several studies also reported autosomal chromosome abnormalities in short stature patients with other clinical features [5-12] and in those without malformations and minor anomalies [12,13].

Chromosomal mosaicism has been known to be associated with the severity of the patient’s phenotype. Patients with mosaic 45,X often have milder symptoms and diagnosed at a later age than patients with nonmosaic 45,X [14] and those with low-level mosaicism may not always be detected by karyotyping thus producing normal karyotyping results. Further analysis on skin fibroblast was performed by Azcona et al. [15] and FISH on buccal smear was suggested by Wolff et al. [16] in Turner syndrome individuals with 46,XX karyotype to detect 45,X mosaicism. Several studies performed interphase FISH and metaphase FISH to detect mosaic 45,X in lymphocytes of nonmosaic 45,X patients [17,18]. Most of previous studies detected other cell population in nonmosaic 45,X karyotyped patients and to the best of our knowledge, confirmation of mosaicism in short stature patients with normal karyotype using FISH technique on uncultured lymphocytes has not been reported. This study presents the cytogenetics findings found in Indonesian patients with short stature. Furthermore, using combination methods of karyotyping and FISH analysis we also reported result discrepancies that have not been reported previously.

## Results

Ninety seven patients were referred due to short stature only or short stature with other clinical features such as dysmorphism, webbed neck, failure to thrive, microcephaly, primary or secondary amenorrhea, cubitus valgus, hypospadia and delayed puberty. Among those patients 88 were females and 9 were males. The age of the patients varied between 5 days to 26 years of age (mean age 10.7 years old).

Eighty patients were karyotyped using conventional G-banding technique, 14 were screened by both karyotyping and FISH and 3 were screened by FISH only due to doctor’s request or the sample did not meet sampling requirement for karyotyping analysis.

The ninety seven patients were divided into two groups, which were Group A consisted of those with other clinical features (40 patients), and Group B those with short stature only (57 patients).

In Group A, twenty two (55%) out of 40 patients had chromosomal abnormalities, of which 45% (18/40) showed sex chromosome abnormalities and 10% (4/40) autosomal abnormalities. In Group B, twenty five (43.85%) out of 57 patients had chromosomal abnormalities, of which 42.1% (24/57) had sex chromosome abnormalities and 1.75% (1/57) had autosomal abnormalities (Table 1).

**Table 1:** Summary of chromosome abnormalities in short stature patients with and without other clinical features based on karyotyping and FISH analysis

**Karyotype and FISH results**	**Group A Short stature patients with other clinical features (no. of cases)**	**Group B Short stature patients without other clinical features (no. of cases)**
46,XX and/or nuc ish (DXZ1x2)	13	29
46,XY	5	3
**Sex chromosomes**		
nonmosaic monosomy X (45,X)	9	8
46,XX and nuc ish (DXZ1x1)//(DXZ1x2)	2	4
46,XY and/or nuc ish (DXZ1x1)//(DXZ1,DYZ3x1)	1	1
mos 45,X/46,XY and nuc ish (DXZ1x1)//(DXZ1,DYZ3x1)// (DXZ1,DYZ3x2)	0	1
mos 45,X/46,XX and nuc ish (DXZ1)//(DXZ1x2)	0	1
46,X,i(Xq)	1	1
mos 45,X/46,X,i(Xq)	2	6
46,X,idic(X)(p22.2) *	0	1
mos 45,X/46,X,idic(X)(q23)	0	1
mos 45,X/46,X,del(Xp)	1	0
mos 45,X/46,X,r(Y)	1	0
mos 45,X/46,X,idic(Y)(pter)	1	0
**Autosomal chromosome**		
mosaic aneuploidy chromosome 18**	1	1
mos 46,XX,add(18p)/ 46,XX	1	0
mos 45,XY,-9/46,XY,r(9)		
(p24q34)		
46,XY,add(11)(q24)	1	0

*FISH result is shown in Table 2.

**consist of one patient (case no. 05 PB25) with mosaic monosomy 18, trisomy 18 and normal, and one (case no. 05 PB73) with mosaic monosomy 18 and normal.

Among those patients in Group A, 8 patients showed mosaic 45,X with 46,XX in 2/40 (5%), XY in 1/40 (2.5%), 46,X,i(Xq) in 2/40 (5%), 46,X,del(Xp) in 1/40 (2.5%), 46,X,r(Y), in 1/40 (2.5%), and 46,X,idic(Y)(pter) in 1/40 (2.5%), whereas among those in Group B, 14 patients showed mosaic 45,X with 46,XX in 5/57 (8.77%), XY in 1/57 (1.75%), XY/XYY in 1/57 (1.75%), 46,X,i(Xq) in 6/57 (10.53%), 46,X,idic(X)(q23) in 1/57 ( 1.75%). Nonmosaic isochromosome Xq (46,X,i(Xq)) was found in 1 patient in each group. Mosaic 46,X,idic(X),(p22.2) was found in Group B.

In our study, 14 patients whom were analysed by both karyotyping and FISH methods showed discrepancies in 10 patients (2 patients from group A and 8 from group B), of which nine of them showed low-level sex chromosomal mosaicism and 1 was mosaic chromosome 18 (Table 2).

**Table 2:** Discrepancy between karyotype and FISH results

**Karyotype**	**FISH [no. of cells]**	**Group A (no. of cases)**	**Group B (no.of cases)**
46,XX	nuc ish (DXZ1x1)//(DXZ1x2)*	2	4
46,XY	nuc ish (DXZ1x1)[77]//(DXZ1,DYZ3)x1[123]	0	1
mos 45,X/46,XY	nuc ish (DXZ1x1)[299]//(DXZ1,DYZ3)x1[65]//(DXZ1x1,DYZ3x2)[36]	0	1
46,X,idic(X)(p22.2)	mos 46,X,idic(X)(p22.2).ish idic(X)(p22.2)(DXZ1+++)[94]//(DXZ1++)[6]	0	1
45,XX,rob(13;14)(q10;q10)	nuc ish (D18Z1x2)[114]//(D18Z1x1)[31]//(D18Z1x3)[5]//(DXZ1x2)[150]	0	1

*Ratio in each patient is shown in Table 4.

**Figure 1:**
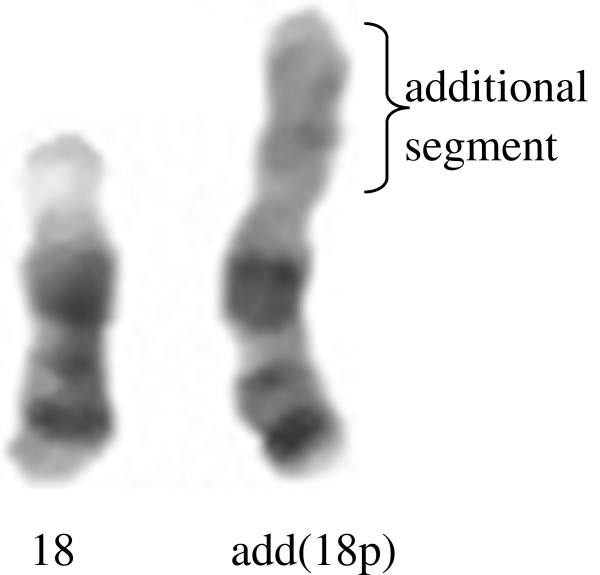
**Partial G-Banded karyotype of chromosome 18.** On the left, is shown the normal chromosome 18; on the right, an additional segment replacing 18p.

**Figure 2:**
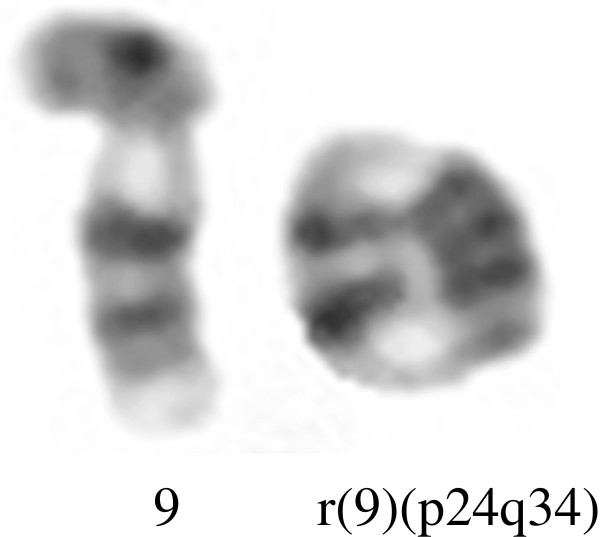
**Partial G-Banded karyotype of chromosome 9.** On the left, is shown the normal chromosome 9; on the right, is the ring chromosome 9.

**Figure 3:**
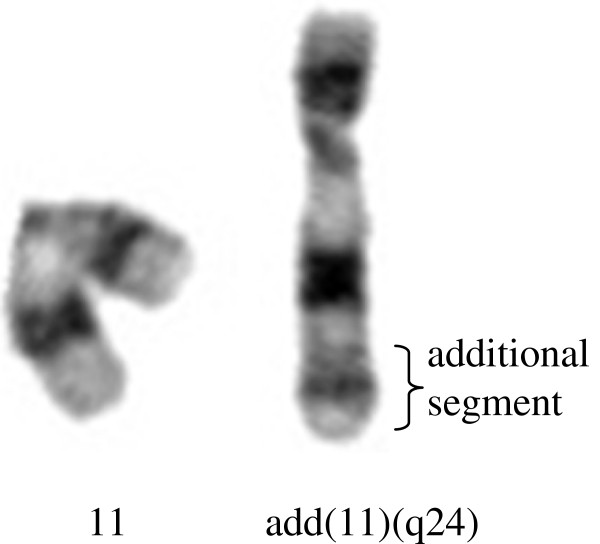
**Partial G-banded karyotype of chromosome 11.** On the left, is shown the normal chromosome 11; on the right, an additional segment attached to band q24 of chromosome 11.

**Figure 4:**
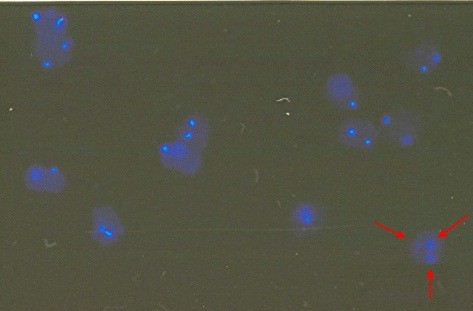
**Interphase FISH result showing mosaic monosomy 18.** The blue signals represent chromosome 18. Three signals: trisomy 18 (red arrows); two signals: diploid number of chromosome 18; one signal: monosomy 18.

**Figure 5:**
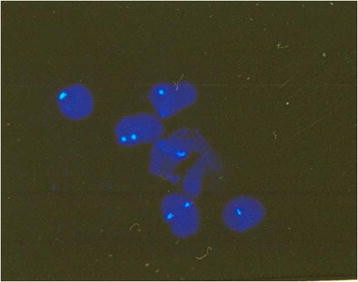
**Interphase FISH result showing mosaic monosomy 18.** The blue signals represent chromosome 18. Two signals: diploid number of chromosome 18; one signal: monosomy 18.

Autosomal chromosome abnormalities were detected in five patients, where two of them had mosaic aneuploidy of chromosome 18 (Case no. 05 PB25 and Case no. 05 PB73), and the other three had mosaic add(18p) (Figure 1), mosaic ring 9 (Figure 2) and add11q24 (Figure 3) (Table 3). One rob t(13;14) karyotyped patient (case no. 05 PB25) showed interphase FISH result of monosomy 18 in 20.7%, trisomy in 18 3.3%, and normal in 76% (Figure 4). The other patient (Case no 05 PB73) showed monosomy 18 in 12% and normal in 88% (Figure 5). In mosaic add(18p) patient (case no. 05 PB53), 46,XX,add(18p) was found in 16% of cell population and 46,XX in 84% (Table 3).

**Table 3:** Short stature patients with autosomal chromosome structural abnormalities

**Case no.**	**Age (yrs)**	**Clinical data**	**Karyotyping results [no. of cells]**	**FISH results [no. of cells]**
05 PB25	12	SS	45,XX,rob(13;14)	Nuc ish (D18Z1x2)[114]//(D18Z1x1)[31]//(D18Z1x3)[5]//(DXZ1x2)[150]
			(q10;q10)	
05 PB53	3	SS, microcephaly, FTT, dismorphic face	mos 46,XX,add(18p)[4]/46,XX[21]	---
05 PB73	12	SS, no menstruation	---	nuc ish (D18Z1x2)[88]//(D18Z1x1) [12]
09 PB42	5	SS, cubitus valgus, clinodactily	mos 45,XY,-9[9]/46,XY,r(9)	---
			(p24q34)[31]	
09 PB443	5	SS, dysmorphism	46,XY,add(11) (q24)	---

*SS* short stature; *FTT* failure to thrive.

**Figure 6:**
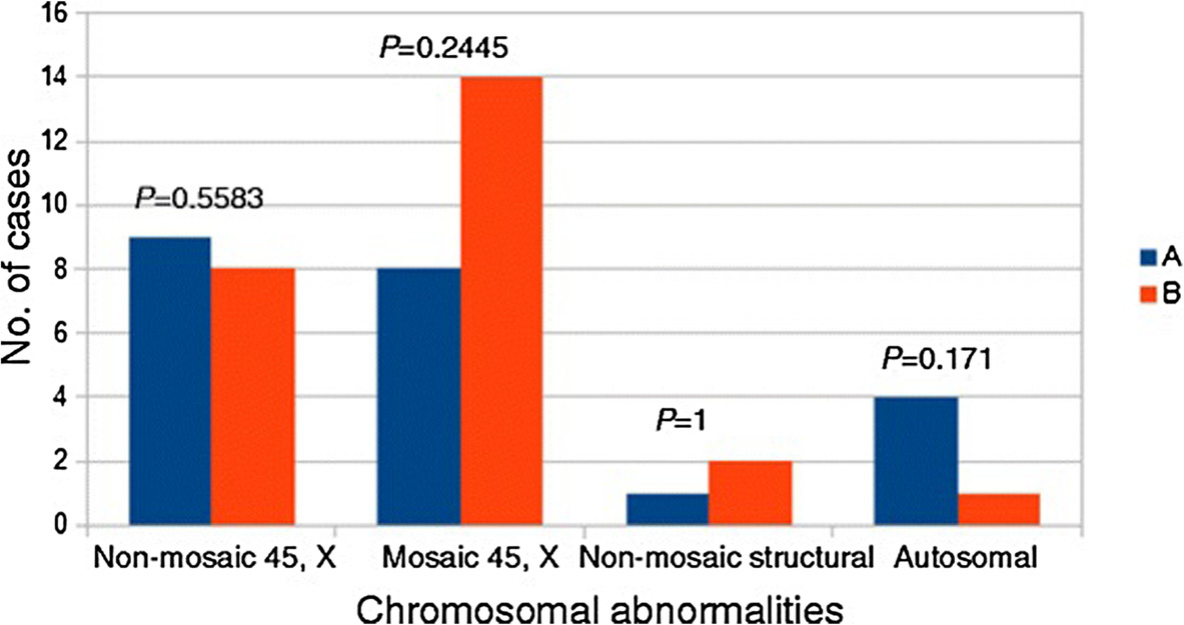
**Total number of cases in each group according to chromsomal abnormalities.***P-values* shown above each group of histograms represent the association betwen chromosomal abnormalities and the phenotypes of the patients. A: group of patients with other clinical features; B: group of patients without other clinical features (short stature only).

Statistical analysis showed no significant association between the groups and the type of chromosomal abnormalities (Figure 6).

## Discussion

The genetic background of short stature have been elucidated by many reports and considerable number of cases can be explained by chromosomal abberations. The incidence of chromosomal anomalies in short stature patients was 2.77% (7/253) in males, in females 9.8% (71/719), and in total 8.02% (78/972), which reported by Moreno-Garcia et al. [19] from a large cohort of patients. Different to our study which resulted in a higher total incidence of 48.45% (47/97) due to selection of patients. Most literatures reported monosomy X is the most common chromosome abnormalities associated with short stature. Having one X chromosome or structural abnormalities of sex chromosome involving the p arm may result in a loss of one copy of SHOX gene, located on each of the X and Y chromosomes in the pseudoautosomal region of the short arms [20], and result in short stature and skeletal abnormalities. Similarly, in our study, this chromosome abberation was found in both groups, which consisted of non mosaic 45,X in 22.5% (9/40), mosaic structural and non-structural in 20% (8/40) and nonmosaic structural 2.5% (1/40) in group A, whereas group B consisted of 14.03% (8/57) non mosaic 45,X, 24.56% (14/57) mosaic structural and non-structural and 3.5% (2/57) nonmosaic structural. Interestingly, autosomal chromosome abnormalities were detected also in both groups (10% (4/40) in group A and 1.75% (1/57) in group B).

**Figure 7:**
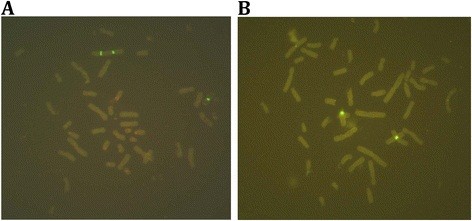
**Metaphase FISH result showing mosaic idic(X)/XX.(A)** shows two green signals in one of the X chromosomes represent idic(X)(p22.2), one green signal represent the normal X chromosome; **(B)** shows one green signal in each of the X chromosome represent the normal X chromosome (46,XX).

To the best of our knowledge, no consensus has been established on cytogenetic grounds for the definition of low-level mosaicism, but some authors considered the presence of aneuploid cells of 6-10% as being low-level mosaicism [21] and <6% by others [22,23]. Our study also showed that low-level mosaicism may occur and this may not be consistantly detected by karyotyping alone. Seven patients showed normal results by karyotyping but by interphase FISH additional cell line of aneuploid cells were detected. Among these patients, six revealed low-level sex chromosome mosaicism, in which 2 of them (case no. 09 PB279 and 09 PB439) showed the presence of monosomy X of less than 10% and 4 (case no. 09 PB422, 09 PB432, 09 PB447, and 09 PB453) showed less than 6% (Table 4). The presence of additional monosomy X cell line, although it is at low level, could be responsible in growth retardation disease. On the other hand, metaphase FISH analysis was carried out in a nonmosaic 46,X,idic(X)(p22.2) patient to confirm isodisentric and the result revealed mosaic idic(X)/XX, of which out of 100 cells analyzed 94 cells (94%) were idicX and 6 (6%) were XX (Table 2 and Figure 7).

**Table 4:** Discrepancies between FISH and karyotyping results in short stature patients with nonmosaic 46,XX karyotyping

**Case no.**	**Group**	**Karyotype**	**FISH results [no. of cells]**
09 PB279	B	46,XX	nuc ish (DXZ1x1)[21]//(DXZ1x2)[229]
09 PB422	B	46,XX	nuc ish (DXZ1x1)[16]//(DXZ1x2)[284]
09 PB432	B	46,XX	nuc ish (DXZ1x1)[10]//(DXZ1x2)[290]
09 PB447	A	46,XX	nuc ish (DXZ1x1)[9]//(DXZ1x2)[291]
09 PB453	A	46,XX	nuc ish (DXZ1x1)[15]//(DXZ1x2)[285]
09 PB439	B	46,XX	nuc ish (DXZ1x1)[23]//(DXZ1x2)[277]

Further analysis with interphase FISH of a mosaic 45,X/46,XY karyotyped patient (Table 2) showed additional XYY cell line (9%) with monosomy X cell line being the most prevalent (74.8%).

The loss of aneuploid cells during cell culture process is considered as the limitation of karyotyping in detecting low level mosaicism. Interphase FISH technique uses uncultured cells thus the chance of finding aneuploid cells is higher. Van Dyke et al. [18] stated that FISH method is sensitive and specific, and can be employed on the same blood sample that is used for the conventional cytogenetic studies.

Wolff et al. [16] mentioned that the detection of a low level of 45,X cells (<10%) during routine cytogenetic analysis of peripheral blood or bone marrow from an adult female can be difficult to interpret. Our patients were in the range of preteens to teenagers (mean age 10.7 years old). According to Russell et al. [24] study the frequency of X chromosome loss at age < 16 years is very low (0.07%), thus the low level of monosomy X in our prepubertal age patients were consistent with Turner syndrome and were not due to age-related X chromosome loss.

In our study interphase FISH analysis were carried out in up to 200 to 300 cells, these large numbers of cells should be sufficient in detecting low-level mosaicsim, as Wiktor et al. [17] suggested that interphase FISH analysis of 200 cells provides a confidence level of 95% at detecting a cell line occurring at 2%.

The discordances between karyotyping and FISH results indicate that FISH analysis should be performed routinely on short stature patients as an adjunct to karyotyping, and metaphase FISH analysis need to be done when patients reveal nonmosaic structural abnormalities of X chromosome to exclude mosaicism. Interphase FISH, however, is a more accurate technique to see ratio of mosaicism than metaphase FISH.

Patients with structural sex chromosome abnormality are mostly tend to be mosaic with 45,X cell line due to the loss of the abnormal chromosome during mitosis [25]. These structurally abnormal sex chromosomes are preferentially inactivated and tend to be lost during subsequent mitotic cell divisions, as shown in long term cultivation experiments, and this finding suggest that the same mechanism also occur in vivo [26]. This mechanism is applied not just for isochromosomes and isodicentric but also for other structural abnormalities such as ring Y and deletion of Xp. This mitotic loss of structurally abnormal sex chromosome may results in the relatively high frequency of mosaicism. This phenomenon was seen in our patients with mosaic isochromosome Xq (45,X/46,X,i(Xq)), which occurred in 8.25% out of 97 patients (2 patients in group A and 6 patients in group B) and was the most frequent structural abnormality found in our series.

The possible mechanism for X/XY/XYY mosaicism is due to post-zygotic mitotic error, which might occur after the first mitosis (Post first cell division nondisjunction). The possible explanation for the low percentage of XY population (16.2%) is there may be further non-disjunction occurred in some of the XY cells resulting in X/XYY cell lines hence lowered the XY cell population and increased the number of cells with monosomy X and XYY, subsequently. The XYY population was too infrequent thus was not detected by karyotyping. This may due to further nondisjunction in XYY or structural abnormalities in one of the Y chromosomes resulting in mitotic instablity. Bulakbasi et al. [27] study reported a Yq microdeletion found in a mos 45.X/46,XY karyotyped patient with mos 45,X/47,XYY/46,XY karyotype from testicular tissue cultures may induce mitotic instability of the Y chromosome and cause mosaicism that may be undetected in blood cells but make considerable contribution to the germinal tissue. Yq microdeletion can be detected using multiplex PCR. Thus we suggest a more sensitive method such as multiplex PCR to discover the mechanism.

Chromosome analysis using both methods showed normal results in three patients diagnosed with short stature (one of them was also diagnosed with dysmorphism). This indicated that the short stature in these patients might be due to other causes rather than chromosomal defect, such as Noonan syndrome, hypothyroidism, celiac disease, lead poisoning, neurofibromatosis, growth hormone deficiency or an underlying medical condition that was previously undiagnosed [2]. Short stature could also be due to constitutional delay of growth and could also be familial.

A single gene disorder such as deletion or mutation of SHOX gene, SHOX (short stature homeobox) could also result in short stature. SHOX gene mutations were more common among children with certain anthropometric and dysmorphic signs [4] and have been detected in 2.4% of children with short stature [20]. However, small deletions or mutations of SHOX gene cannot be detected by conventional chromosome analysis. Therefore, further study to detect SHOX gene mutation is suggested in short stature patients with normal karyotype and FISH results. As suggested by ACMG practice guideline [28], for short stature patients with other anomalies/malformations that are consistent with recognizable genetic syndrome molecular genetic testing, including SHOX gene testing, is required to confirm the condition. If the anomalies/malformations do not suggest a recognizable syndrome genomic array studies may be considered to evaluate any changes of genomic copy number. Molecular genetic evaluation including genomic array studies are required to confirm the condition in those without chromosomal defect, although to most of Indonesian population the cost of such genetic evaluation is still considered expensive.

Autosomal chromosome abnormalities have also been known to be associated with short stature [5-13]. In our study, a normal karyotype patient (Case no. 05 PB25) was found to have mosaic aneuploidy of chromosome 18, of which 20.7% were monosomy 18, 3,3% were trisomy 18 and 76% were normal using interphase FISH (Table 4), while interphase FISH result of another patient (Case no. 05 PB73) showed mosaic monosomy 18 (12%) and normal (88%). To lower the incidence of missed-interpretation of probes and karyotyping, all FISH and karyotyping analysis were performed by two certified cytogeneticists. Although, when it is available, a more sensitive method should be performed. These findings cannot be ignored and must be confirmed by further analysis.

There are several possible mechanisms for mosaic aneuploidy of chromosome 18. Firstly, due to non-disjunction that may occur after the first mitosis of post-zygotic cell division resulting in mosaic with 46 chromosomes (diploid) from the first mitosis and with 45 (monosomy) and 47 chromosomes (trisomy) from the second mitosis (this might be the mechanism for Case no. 05 PB25), while anaphase lag is the suggested mechanism for the formation of mosaicism (monosomy and diploid) in Case no. 05 PB73. Secondly, the presence of ring chromosome 18, which is unstable and tends to be lost in mitosis, resulting in mosaic aneuploidy chromosome 18. Cells with one copy of the chromosome are more often severely disadvantaged and usually will not continue to reproduce. This explains the lower ratio of monosomy population compared to normal (diploid).

Ring chromosomes are formed due to breakage at both ends of the p and q arms and the joining back of the broken ends with loss of terminal regions. Loss of 18q region has been identified in growth hormone deficiency (GHD) patients and out of 60 genes contained in this region, 2 genes have been identified and one of these genes, the galanin receptor type 1(GALR1) which is a candidate gene for GHD due to its hypothalamic involvement in growth hormone regulation [5]. GALR1 gene is located in 18q23, which is at the terminal of long arm of chromosome 18. Maranda et al. [7] revealed 46,XX,del(18)(p11.2) in patients with short stature, dysmorphic features and moderate mental retardation. Further investigation using FISH with 18p/18q subtelomeric probes showed a terminal deletion of 18p. Thus terminal deletion of 18p and 18q is associated with short stature. The loss of terminal regions in ring chromosome 18 and the loss of GALR1 gene cause short stature. Clinically, in this case, hormonal profile is needed to confirm growth hormone deficiency.

In case no. 05 PB53, an additional segment of unknown origin replaced the whole p arm of chromosome 18 by attaching to the centromere. The loss of the whole p arm of chromosome 18 is associated with short stature, microcephaly and dysmorphic features [11]. Despite these clinical findings other dysmorphologies may be manifested as the consequences of active genes contained in the additional segment. However, the severity of the clinical features is also determined by the ratio of the mosaicism, since the karyotyping showed mosaic 46,XX,add(18p)[16%]/46,XX[84%] which is shown in Table 3.

In the case of additional of unknown segment, parental karyotyping should be carried out to discover its origin, as most of such cases are the result of balance translocation that occurred in one of the parents. Unfortunately, in both of our patients with additional segment (case no. 05 PB53 and 09 PB438) parental karyotypings were not carried out. For the case of normal parental karyotype, structural mosaicism is most likely occured spontanously during early mitosis of an initially normal zygote, in which it is relatively a rare incidence [29].

In case no. 09 PB438, an additional segment was attached to band q24 and replaced the segment q25 and the terminal region of chromosome 11. 11q terminal deletion disorder is also known as Jacobsen syndrome, in which among other clinical manifestations, also causes short stature [10,30]. Most children with this syndrome are reported to have IGF-I (insulin growth factor-I) deficiency [10].

Short stature patients with ring chromosome 9 have been reported [9,13]. Terminal deletions of 9p and 9q are associated with growth retardation [9]. The ring structure is unstable therefore it tends to be eliminated during mitosis resulting in mosaic 45,XY,-9/46,XY,r(9)(p24q34).

Besides FISH technique, comparative genomic hybridization or SNP array are now widely used to eliminate culture bias produced by analysis of metaphase cells by analyzing DNA extraction from both interphase and metaphase cells and to detect low-level mosaicism by simultaneous evaluation of DNA copy number to detect mosaic gains and losses [31]. Further analysis using this technique should be performed to confirm patients with low-level mosaicism and discordant results.

Statistical analysis showed no significant association between the groups and the type of chromosomal abnormalities. This finding suggests that short stature patients without other clinical features could have similar opportunities of carrying mosaic or other chromosomal abnormalities as those with other clinical features. This indicates chromosome analysis is required for short stature evaluation, especially in patients without clear clinical etiologies.

In conclusions, wide variations of chromosome abberations were found in short stature patients with and without additional clinical features. Exlcuding nonmosaic monosomy X, our study showed chromosome abnormalities were detected in 48.45% of short stature patients with and without other clinical features, and the abnormalities include non-structural sex chromosomes mosaicism, mosaic and nonmosaic sex chromosomes structural abnormalities and autosomal structural abnormalities. Interphase FISH needs to be routinely performed as an adjunct to karyotyping to detect low-level mosaicsm in short stature patients. Although it is rare, autosomal chromosome abnormalities which mostly involving terminal regions of certain chromosomes, can be responsible for growth failure. Thus subtelomeric FISH is suggested to be carried out in such cases. However, it is now known that genomic array/SNP array technology, as one of the most recent technologies for genetic evaluation, is a more sensitive method and can easily detect subtelomeric deletions and mosaicism.

So far no data of short stature patients in Indonesian population has been established based on cytogenetic findings. This study provides recent data of chromosomal background in short stature patients, especially in Indonesian population.

## Materials and methods

### Subjects

The subjects in this study were short stature patients (less than 3rd percentile) and short stature with other clinical features. The patients were referred by clinicians all over Indonesia to Cytogenetics laboratory at the Eijkman Institute during the year 2003–2009.

### Karyotyping

Standard Karyotyping analysis was performed, using metaphase chromosome preparations from phytohemagglutinin stimulated blood lymphocytes of the peripheral blood [32]. The metaphases were stained using G-banding technique [33]. Following metaphase staining chromosome analysis were carried out. For each patient a total of 20 metaphases were analyzed, in which 15 metaphases were counted and 5 were counted and structurally analyzed. When mosaicism present a total of 40 metaphases were analyzed, in which 35 metaphases were counted and 5 were counted and structurally analyzed. The analysis and nomenclature of the chromosomes were based on ISCN 2009 (An International System for Human Cytogenetic Nomenclature (2009) [34].

### Fluorescence In Situ Hihybridization (FISH)

Standard interphase fluorescence in situ hybridization technique were applied on uncultured cells to confirm the ratio of mosaicism, using CEP X SpectrumGreen/ CEP Y SpectrumOrange (Vysis) dual colour probe specific to the centromere of human chromosome X (bands Xp11.1-q11.1, locus DXZ1) and to the centromere of human chromosome Y (bands Yp11.1-q11.1, locus DYZ3), respectively. Metaphase FISH was carried out to confirm structurally abnormal sex chromosomes. Chromosome 18 interphase FISH was carried out using CEP 18 SpectrumAqua (Vysis), which specific to the centromere of human chromosome 18 (band region 18p11.1-q11.1, locus D18Z1).

The post-hybridized interphase and metaphase were counterstained with 125 ng/ml DAPI II suspended in an antifade solution (Abott Molecular Inc.). A minimum of 50 post-hybridization interphases or metaphases were analyzed using Nikon eclipse 80i microscope with D-FL Epi-Fluorescence attachment. The images were captured by Nikon DS Cooled camera and examined using NIS Elements Basic Research imaging software. 50 interphases or metaphases were analyzed but for those where mosaicism present up to 300 interphases or metaphases were analyzed.

To lower the incidence of missed-interpretation of probe signals and karyotyping, all FISH and karyotyping analysis were confirmed by at least two certified cytogeneticists.

## Statistical analysis

Statistical analysis was carried out to determine the association between the groups and the type of chromosomal abnormalities. It was performed by Fisher Exact test and *P-*values of less than 0.05 was considered significant.

## Competing interests

The authors declare that they have no competing interests.

## Authors’ contributions

CP performed the karyotyping and FISH analysis, collected and analyzed the data, drafted, revised, and finalized the manuscript. HK performed the karyotyping and FISH analysis and helped to draft the manuscript. DDA, HWA and LPS carried out karyotyping and FISH analysis. NSM counseled the patients, helped to draft and involved in revising the manuscript. ARH supervised the cytogenetic results, involved in manuscript revision and gave the final approval of the manuscript. All authors read and approved the final manuscript.
